# Retraction: Potassium fertilization improves growth, yield and seed quality of sunflower (*Helianthus annuus* L.) under drought stress at different growth stages

**DOI:** 10.1371/journal.pone.0272194

**Published:** 2022-08-03

**Authors:** The PLOS ONE Editors

The *PLOS ONE* Editors retract this article [1] because it was identified as one of a series of submissions for which we have concerns about authorship, competing interests, and peer review. We regret that the issues were not addressed prior to the article’s publication.

MAC, MAB, and MJA did not agree with the retraction. JSD, MIAR, SK, SR, ALV, SH, SMA, FMAZ, and KH either did not respond directly or could not be reached.
